# Anais Brasileiros de Dermatologia: who wrote this century-old history?^☆^

**DOI:** 10.1016/j.abd.2025.02.001

**Published:** 2025-03-17

**Authors:** Helena Cargnelutti Grimaldi, Sandro da Silva Camargo

**Affiliations:** a Private Clinic, Bagé, RS, Brazil; b Graduate Program in Applied Computing, Federal University of Pampa, Bagé, RS, Brazil

**Keywords:** Authorship, Bibliometrics, Periodical, Scientific communication and diffusion

## Abstract

The *Anais Brasileiros de Dermatologia* (ABD), an official publication of the Brazilian Society of Dermatology, has been published since 1925. ABD is considered the most influential dermatological journal in Latin America. By 2025, the journal will mark a significant milestone, celebrating a century of history. Over this time, it has published 99 volumes and 6,299 contributions from more than 10,800 authors. To analyze the trajectory of the journal, this study employs an applied research approach characterized by descriptive objectives and a quantitative nature. This research was based on a documentary procedure that encompassed all contributions already published in the journal. The main goal of the work was to identify the most prominent authors who have contributed to the journal and to map the largest co-authorship communities. The authors hope that this research serves as a formal recognition of the researchers who have written the history of the ABD.

## Introduction

The *Anais Brasileiros de Dermatologia* (ABD) is the authoritative scientific publication endorsed by the Brazilian Society of Dermatology. ABD stands out as one of the leading specialized journals in the field of dermatology, garnering recognition not only within Brazil but also on an international scale.^1^ Established in 1925, the journal has remained steadfast in its commitment to disseminating scientific knowledge and fostering the progress of dermatology. Covering a diverse array of topics concerning skin, hair, and nail health, ABD continues to be a pivotal resource in advancing the understanding of dermatological sciences. Reaching the milestone of a century of contributions prompts reflection on the past and recognition of the researchers who have helped write this success story. Moreover, with 99 volumes published, the journal chronicles the evolution of dermatology in Brazil.

Analyzing the history of a journal through significant milestones is a common practice in the literature. The Journal of Dental Research underwent a bibliometric analysis covering its 100-year history. The study identified the 100 most cited articles from this period. The findings highlight the journal’s extensive impact and reveal shifts in citation patterns and research priorities over the past century.^2^ Similarly, the Journal of Prosthetic Dentistry was subjected to a comprehensive bibliometric analysis to examine its characteristics over a 50-year period, from 1970 to 2019. Of the 11,989 records retrieved, 10,638 (92.9%) were included in the analysis.^3^ Likewise, during its 20-year history, the *Centro de Ciências da Economia e Informática* (CCEI) Journal was analyzed, which includes all 420 published articles. The study identified the most influential authors and their associated research communities.^4^ Analogously, the *Arquivos Catarinenses de Medicina* Journal was analyzed, covering all 1,173 articles published since its inception 65 years ago. The study identified the most prominent authors and their primary research communities.^5^ To assess the current status and research trends in caries diagnosis, 816 documents published between 2013 and 2021 were analyzed from the Web of Science Core Collection database. The study aimed to perform a bibliometric analysis to identify contributing researchers, organizations, countries or regions, and journals, as well as to examine keyword co-occurrence and co-authorship networks.^6^

In parallel, other studies use Social Network Analysis (SNA) to obtain a deeper insight into the structure of collaboration among the authors. In medical education, it is acknowledged that SNA is underutilized. However, the method is argued to have significant potential, offering valuable insights that could enhance the experiences and outcomes of medical trainees and educators, ultimately benefiting patients.^7^ SNA was also employed to visualize the co-authorship networks and scientific map of research outputs in clinical teaching and medical education. The research examined 1,229 publications on clinical teaching spanning a 40-year period, from 1980 to 2018.^8^

The ABD itself has been the subject of studies involving importance metrics, but the studies focused on journal impact indicators, without addressing aspects related to author metrics. An analysis of the trajectory of the ABD over a decade was done, from 2013 to 2022, and compared key bibliometric indices with those of Brazilian medical and international dermatological journals.^9^ Previously, trends in the main bibliometric indicators of the ABD from 2010 to 2019 were investigated.^10^

In this context, the aim of this research is to analyze the century-long history of the ABD, identifying its most prominent contributors and mapping the largest co-authorship communities.

## Material and methods

This study is an applied research with a descriptive objective and a quantitative nature. It involved a documentary procedure conducted on the websites of the ABD. This section delineates the methods employed in the study, encompassing the data collection process, the creation of the database, the generation of the co-authorship network, and the calculation of author metrics.

### Dataset

Data was collected through the web scraping technique, which enabled the extraction of data from websites.^11^ To automate this process, a scraper was implemented to download data from all published editions available on the journal’s old website (http://www.anaisdedermatologia.com.br/edicoes-anteriores). The scraper was executed on December 24, 2024, at 15:56, performing a complete copy of the site’s public data at that time. Data from volume 1, issue 1 (1925) to volume 95, issue 6 (2020) were downloaded, covering 96-years of the journal’s history. The data from the Journal’s new website (https://www.sciencedirect.com/journal/anais-brasileiros-de-dermatologia/issues) were manually downloaded, covering from volume 96 issue 1 (2021) to volume 99 issue 6 (2024). These steps involved the download of 99 volumes, encompassing a total of 6,299 contributions, which includes case reports, reviews, editorials, and full articles.

Afterward, the downloaded data underwent preprocessing to extract the names of the authors from each work, identifying 22,200 authors. A common issue in determining the impact is duplicate names, may arise from inconsistencies in the names used or name changes.^12^ In this sense, the third step involved employing the Levenshtein algorithm (https://www.rdocumentation.org/packages/utils/versions/3.6.2/topics/adist) to calculate the distance between names, revealing several authors whose names had been written differently. For example, author Rubem David Azulay had his name written in the following alternative ways: R. D. Azulay, Dr. Rubem D. Azulay, Rubem D. Azulay, and Ruben D. Azulay. After standardizing the authors’ names, the number of distinct authors was reduced from 22,200 to 10,829. This protocol for standardizing names has been applied in similar studies.^5^, ^13^

### Computing authors relevance

Finally, the collaboration network graph was generated, and author metrics were computed using the Gephi tool (https://gephi.org/). The concept of graphs is fundamental for understanding SNA. A graph is an abstract representation of a set of objects and their relationships.^14^ In this work, the objects are the authors, and the relationships are co-authorship interactions. Key concepts of graphs for SNA include: 1) Nodes represent each author who has published in the journal; 2) Edges represent co-authorship relationships that occurred in the same paper; 3) Graph represents the co-authorship structure among all authors in the journal over its 99-years; 4) The graph is of the Undirected type, as the order of authors in each article was not considered; and 5) Edge Weights represent the number of co-authorships between two linked authors.

In this study, based on graph theory,^14^ which serves as the theoretical foundation for SNA, the following metrics were calculated for the authors:

• Number of Publications (Pub): This metric reflects the total number of publications in which the author has participated, regardless of their position among the co-authors.
• Degree (Deg): This metric indicates how many different authors collaborated in co-authoring works with the author. The importance of the degree in a co-authorship network lies in its ability to reveal the collaborative behavior and networking patterns of authors within a research community. High-degree authors are often well-connected hubs within the research community and may have significant influence over the flow of information, ideas, and collaborations within their field.^15^
• Betweenness Centrality (BC): This metric indicates the relevance of an author as a connection between different research groups.^16^ Elements with high betweenness centrality can be considered key players or influencers within the healthcare system.^17^ These nodes may represent hospitals, healthcare providers, or diseases that play a crucial role in the dissemination of information, patient referrals, or the spread of diseases. This metric is also recognized as a robust measure for identifying the most relevant genes, making them potential candidates for drug-targeting purposes.^18^, ^19^
• Page Rank (PR): Developed by Google, this metric was designed to determine the order in which web pages are displayed to users during searches. Recently, this metric has been used for identifying influential researchers in citation networks.^20^, ^21^ In the present work, PR is utilized to identify authors who hold leadership positions within the community of ABD.
• Community (Com): The identification of co-authorship communities was conducted using the Louvain method.^22^ The community number indicates its position in the ranking of the largest communities, such that community 1 is the largest in terms of number of members, community 2 is the second largest, and so forth.

## Results and discussion

Table 1 displays the authors who had at least thirty-one publications in the ABD. This threshold enabled the selection of the top 50 authors with the highest number of publications, representing 0.46% of the journal’s 10,829 authors. The total number of publications by the top 50 authors is 2,550, out of a total of 6,299 publications by ABD up to its 99^th^ edition. These numbers highlight the importance of the top 50 authors.

**Table 1:** Top 50 authors ranking.

Pos	Name	Pub	Deg	BC	PR	Com
1	Rubem David Azulay	**161**	(2) **184**	(2) **0.043302**	(3) **0.002593**	1
2	Helio Amante Miot	**143**	(1) **266**	(1) **0.048551**	(1) **0.003699**	5
3	Silvio Alencar Marques	**105**	114	0.017461	(5) **0.002118**	5
4	Hiram Larangeira de Almeida Junior	**99**	(5) **160**	0.018632	(2) **0.002874**	3
5	Renan Rangel Bonamigo	**87**	(3) **179**	0.024416	(4) **0.002485**	3
6	Bernardo Gontijo	78	95	(4) **0.030107**	0.001530	8
7	Neusa Yuriko Sakai Valente	76	133	0.012516	0.001980	6
8	Nelson Guimaraes Proenca	76	58	0.004664	0.001008	11
9	Juan Manuel Pineiro Maceira	63	158	0.019992	0.001939	1
10	Sinesio Talhari	62	100	0.022246	0.001561	10
11	Everton Carlos Siviero do Vale	61	56	0.007561	0.001060	8
12	Paulo Ricardo Criado	60	128	0.019865	0.001789	6
13	Antonio de Souza Marques	56	97	0.020951	0.001427	1
14	Nurimar Conceicao Fernandes	53	101	0.006272	0.001497	1
15	Antar Padilha Goncalves	50	9	0.000759	0.000246	13
16	Mariangela Esther Alencar Marques	49	93	0.003028	0.001346	5
17	Antonio Pedro Mendes Schettini	48	84	0.005235	0.001350	10
18	Luiz Carlos Cuce	48	63	0.010051	0.001027	23
19	Milvia Maria Simoes e Silva Enokihara	47	101	0.009096	0.001413	15
20	Sebastiao A Prado Sampaio	47	43	0.005636	0.000575	23
21	Luna Azulay Abulafia	44	(4) **162**	(3) **0.038265**	0.001622	2
22	Izelda Maria Carvalho Costa	44	63	0.005288	0.000956	12
23	Lucia Martins Diniz	43	55	0.005028	0.001120	1
24	Tancredo Alves Furtado	42	45	0.007044	0.000654	8
25	Glyne Leite Rocha	42	40	0.001813	0.000705	1
26	Alexandre Carlos Gripp	41	112	0.016335	0.001218	2
27	Leninha Valerio do Nascimento	41	67	0.005549	0.000769	2
28	Rosana Lazzarini	39	91	0.006285	0.001070	7
29	Absalom Lima Filgueira	38	50	0.008273	0.000664	1
30	Tania Ferreira Cestari	37	144	0.021105	0.001482	4
31	Fabiane Andrade Mulinari Brenner	37	89	0.012498	0.001173	9
32	David Rubem Azulay	36	85	0.010202	0.001165	2
33	John Verrinder Veasey	36	67	0.002917	0.000891	7
34	Lucio Bakos	35	81	0.008165	0.001021	3
35	Luciana Patricia Fernandes Abbade	35	64	0.005179	0.000892	5
36	Omar Lupi da Rosa Santos	34	94	0.015268	0.001058	1
37	Antonio Carlos Pereira Junior	34	61	0.004186	0.000812	1
38	Francisco Eduardo Rabello	34	16	0.000398	0.000183	1
39	Demetrio Peryassu	34	11	0.000437	0.000289	13
40	Juliano Vilaverde Schmitt	33	83	0.009619	0.000976	5
41	Vitor Manoel Silva dos Reis	33	72	0.005796	0.000837	11
42	Helena Muller	33	66	0.008979	0.000864	11
43	Elemir Macedo de Souza	33	50	0.006729	0.000724	18
44	Lorivaldo Minelli	33	39	0.003786	0.000732	21
45	Mario Cezar Pires	32	112	0.013097	0.001184	4
46	Carolina Talhari	32	76	0.022741	0.001040	10
47	Monica Santos	32	53	0.002048	0.000908	10
48	Itamar Belo dos Santos	32	49	0.003673	0.000917	14
49	Carlos Baptista Barcaui	31	84	0.010410	0.000937	1
50	Adriana Maria Porro	31	72	0.005599	0.000890	15

Pos, Position; Pub, Publications; Deg, Degree; BC, Betweenness Centrality; PR, PageRank; Com, Community.

Table 1 is sorted in descending order of Publications, Degree, Betweenness Centrality, and Pagerank, indicating the respective co-authorship communities. In the Deg, BC, and PR columns, the values in parentheses indicate the author’s potential ranking position if the respective column were used as the primary sorting criterion. In addition to these authors presented in the ranking, 57 authors were identified who had twenty or more articles published, 169 authors with ten to nineteen articles, 397 authors with five to nine articles; 2,182 authors with two to four articles, and 7,974 authors who participated in a single article, indicating that 73.64% of the authors published only once in the journal.

Due to space limitations, the authors chose to restrict the analysis to the top 5 highest values of each metric. Thus, according to number of publications, the importance over the 99-years of ABD is evident for the following five authors: Rubem David Azulay (Pos = 1) with 161 publications, Helio Amante Miot (Pos = 2) with 143 publications, Silvio Alencar Marques (Pos = 3) with 105 publications, Hiram Larangeira de Almeida Junior (Pos = 4), with 99 publications, and Renan Rangel Bonamigo (Pos = 5), with 87 publications. Just these authors contributed 595 publications, accounting for over 9.44% of the journal’s total publications throughout its history. The median number of articles published by the top 50 ranked authors was 41.5 (p25–p75: 34–56), indicating a highly productive subgroup. In contrast, the overall average number of publications per author was much lower, 2.05 ± 4.59, with a median of 1 (p25–p75: 1–2). This substantial disparity suggests an exponential distribution, where a small number of highly productive authors contributed substantially to the total publication output.

In terms of degree, which represents the number of different co-authors collaborated with, the following authors stand out: Helio Amante Miot (Pos = 2) with 266 co-authors, Rubem David Azulay (Pos = 1) with 184 co-authors, Renan Rangel Bonamigo (Pos = 5) with 179 co-authors, Luna Azulay Abulafia (Pos = 21) with 162 co-authors, and Hiram Larangeira de Almeida Junior (Pos = 4) with 160 collaborations. The median number of coauthors among the top 50 ranked authors was 82 (p25–p75: 56–101). In comparison, the average number of coauthors across all authors was 6.2 ± 9.29, with a median of 4 (p25–p75: 3–6). This disparity highlights the more extensive collaborative networks of the top authors compared to the broader author pool.

The highest Betweenness Centrality values indicate authors who played a significantly relevant role in communication between different research communities, thereby integrating various communities within the context of ABD. In this metric, the following authors are highlighted: Helio Amante Miot (Pos = 2), Rubem David Azulay (Pos = 1), Luna Azulay Abulafia (Pos = 21), Bernardo Gontijo (Pos = 6), and Cesare Massone (Pos = 283). Despite a lower number of publications (Pub = 9), Cesare Massone, from Galliera Hospital in Genoa, Italy, achieves a high BC score due to his co-authorships with researchers from Brazil, Italy, and Austria. The median BC among the top 50 ranked authors was 0.008219 (p25–p75: 0.005179–0.017461). In contrast, the average BC across all authors was 0.000187 ± 0.001354 (p25–p75: 0–0). This significant difference underscores the central role of the top authors in connecting various segments of the research network.

The highest Pagerank values show the authors who have a leadership role in the ABD, likely resulting from their activities as mentors of new researchers in this area. In this metric, the most relevant authors are: Helio Amante Miot (Pos = 2), Hiram Larangeira de Almeida Junior (Pos = 4), Rubem David Azulay (Pos = 1), Renan Rangel Bonamigo (Pos = 5), and Silvio Alencar Marques (Pos = 3). The median PageRank (PR) among the top 50 ranked authors was 0.001049 (p25–p75: 0.000864–0.001482). In contrast, the mean PageRank across all authors was 9.23 × 10^-5^ ± 1.18 × 10^-4^.

Relationships between these metrics provide insights into the profile of top-ranked authors. A weak positive correlation (p = 0.474) is observed between the number of Publications (Pub) and the number of unique coauthors (Deg). The Deg/Pub ratio among the top 50 authors varies widely, ranging from 0.18 to 3.89, with a median of 1.77 (p25‒p75: 1.29‒2.32). On one hand, Antar Padilha Gonçalves (Pos = 15) has a Deg/Pub ratio of 0.18 (9 coauthors/50 publications), reflecting his tendency to publish primarily without coauthors. A similar pattern is observed with Demetrio Peryassu (Pos = 39), whose ratio is 0.32. This profile is characteristic of authors who were more productive during the earlier decades of ABD and are often regarded as influential figures in the history of Brazilian dermatology.^23^, ^24^ On the other hand, Tania Ferreira Cestari (Pos = 30) has a Deg/Pub ratio of 3.89, indicating collaboration with a diverse range of coauthors in her publications. As another example, Luna Azulay Abulafia (Pos = 21) has a ratio of 3.68. Higher ratios are more characteristic of authors with recent productivity, highlighting an increasing trend in interconnection and collaboration among researchers over time.

A weak positive correlation (p = 0.397) was observed between a number of publications and Betweenness Centrality (BC). The median BC/Pub ratio is 0.18 × 10^-3^ (p25‒p75: 0.12 × 10^-3^ – 0.33 × 10^-3^). Francisco Eduardo Rabello (Pos = 38) has the lowest BC/Pub ratio at 0.011 × 10^-3^, while Luna Azulay Abulafia (Pos = 21) exhibits the highest ratio at 0.869 × 10^-3^. This comparison further highlights the discrepancy between older and more recent authors. In contrast, a very strong positive correlation (p = 0.814) is observed between Deg and BC, reflecting the strong interdependence between a researcher’s collaborative reach and their role as a connector within the network.

When analyzing the relationship between the number of Publications (Pub) and Page Rank (PR), a moderate positive correlation (p = 0.636) is identified. The median PR/Pub ratio is 0.26 × 10^-3^ (p25‒p75: 0.20 × 10^-3^ – 0.29 × 10^-3^). Antar Padilha Gonçalves (Pos = 15) has the lowest PR/Pub ratio at 0.049 × 10^-3^, while Tania Ferreira Cestari (Pos = 30) exhibits the highest ratio at 0.40 × 10^-3^. In contrast, a very strong positive correlation (p = 0.919) is observed between Deg and PR. This indicates that researchers with a broader collaborative network tend to have higher PageRank values, reflecting their centrality and influence within the co-authorship network. The strong relationship underscores the importance of collaborative reach in determining a researcher’s prominence and visibility in the network.

**Figure 1:**
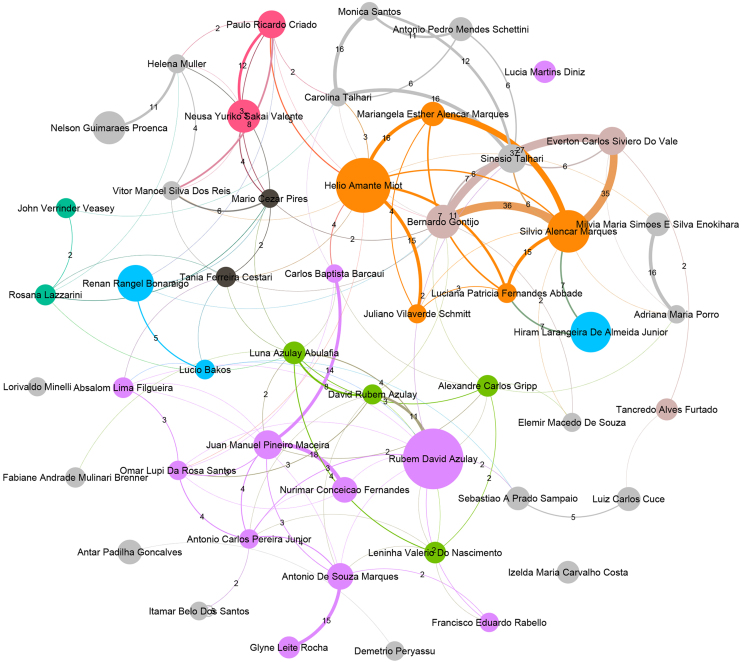
Top 50 authors and their co-authorship networks.

Fig. 1 represents authors in the top 50 ranking and their co-authorships. This figure represents the core of ABD. Nodes in the graph represent authors, with their size proportional to the number of publications. Edges represent co-authorship collaborations, and their thickness indicates the frequency of these collaborations. Higher-weight edges often highlight frequent partnerships, suggesting long-term collaborations, joint projects, or shared research interests. A total of 33,596 co-authorship partnerships were identified, with the most frequent collaborations depicted in Fig. 1. The most frequent co-authorship was observed between Bernardo Gontijo (Pos = 6) and Everton Carlos Siviero do Vale (Pos = 11), with 37 joint publications. Additionally, Silvio Alencar Marques (Pos = 3) exhibited strong collaborative ties with both Bernardo Gontijo and Everton Carlos Siviero do Vale, co-authoring 36 and 35 publications, respectively. In summary, 43 co-authorships were recorded more than 10 times, while 3,655 co-authorships occurred more than once. Notably, nearly 89% of the collaborations, totaling 29,898, took place only once.

The colors of the nodes denote the primary coauthorship communities, with unique colors assigned to the eight largest communities. Communities ranked ninth and smaller are uniformly represented in gray.^5^ These communities typically reflect specialized research areas or institutional affiliations. Notably, communities 1, 5, 10, and 2 together account for nearly half of the top 50 ranked authors, with 11, 5, 4, and 4 members, respectively.

**Figure 2:**
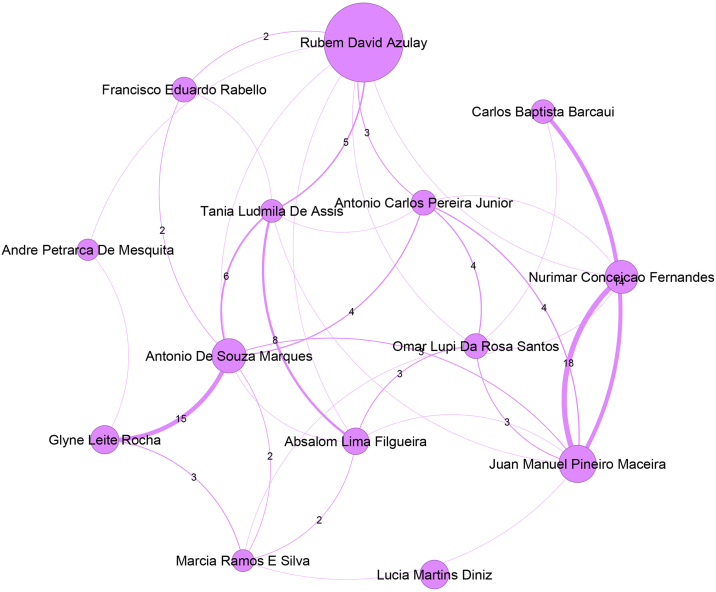
Top authors of the largest community, which contains 831 authors, are predominantly associated with the Universidade Federal do Rio de Janeiro.

**Figure 3:**
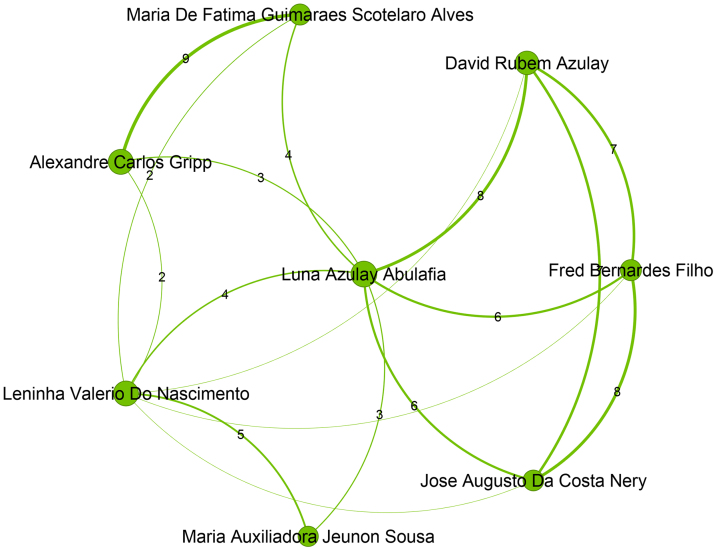
Top authors of the second largest community, which contains 549 authors, are mainly filiated with Universidade Estadual do Rio de Janeiro.

**Figure 4:**
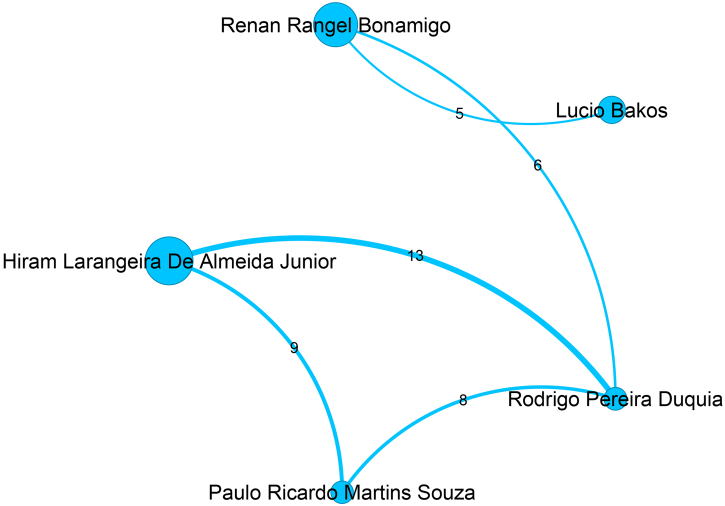
Top authors of the third largest community, which contains 534 authors, are mainly filiated with Universidade Federal do Rio Grande do Sul, Universidade Federal de Ciências da Saúde de Porto Alegre, and Universidade Federal de Pelotas.

**Figure 5:**
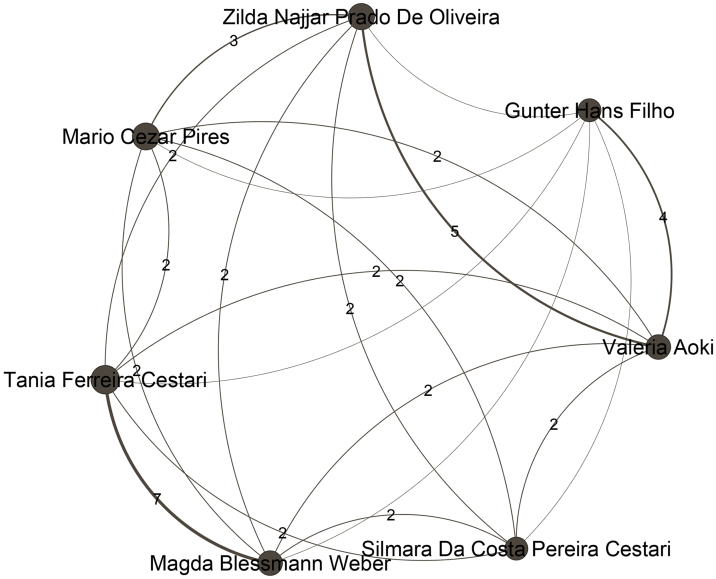
Top authors of the fourth largest community, comprising 350 authors, are associated with different institutions: Universidade Federal de São Paulo, Universidade Federal do Rio Grande do Sul, Universidade Federal do Mato Grosso do Sul, Universidade Federal de Ciências da Saúde de Porto Alegre, Hospital Sírio Libanês, and Hospital do Servidor Público Estadual de São Paulo.

**Figure 6:**
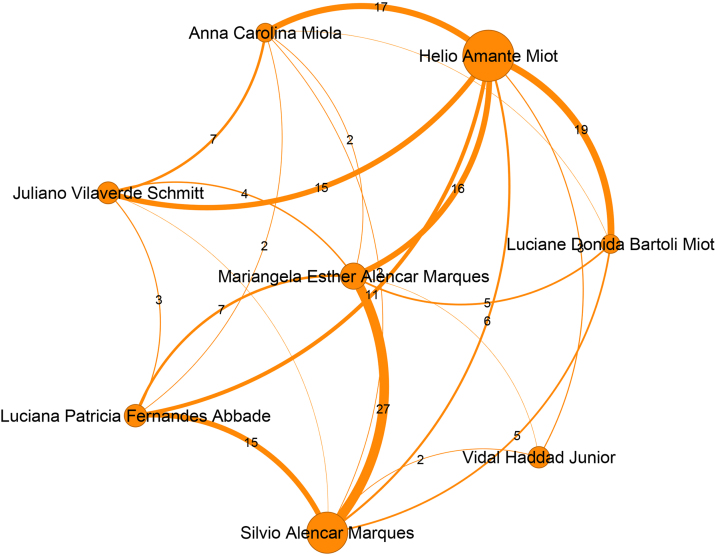
Top authors of the fifth largest community, holding 349 authors, are associated with Universidade Estadual Paulista.

**Figure 7:**
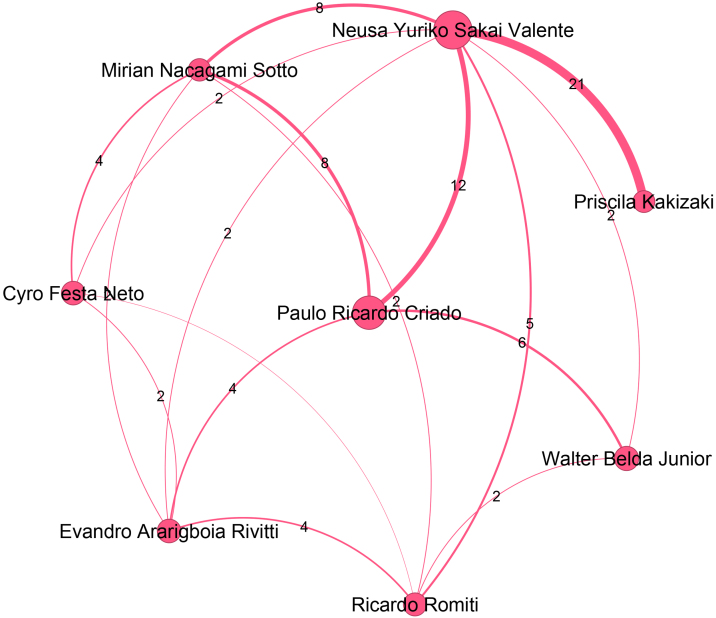
Top authors of the sixth largest community, consisting of 334 authors, are predominantly affiliated with the Universidade de São Paulo.

**Figure 8:**
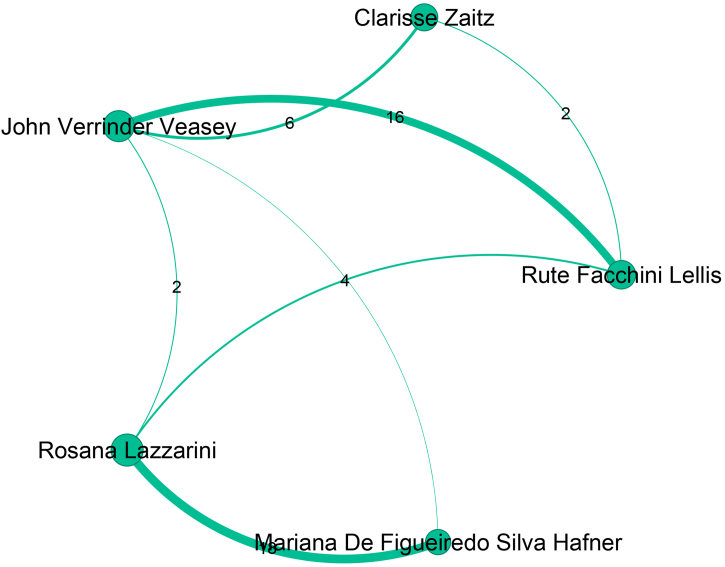
Top authors of the seventh largest community, which contains 320 authors, are primary affiliated with the Santa Casa de São Paulo.

**Figure 9:**
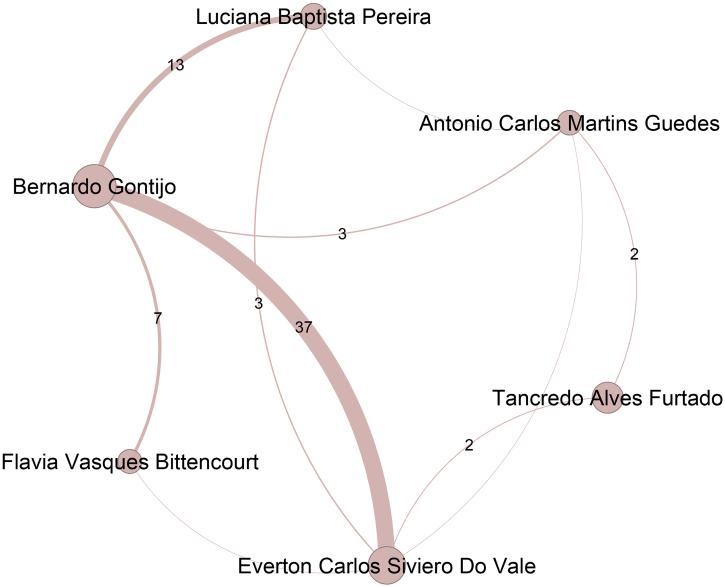
Top authors belonging to the eighth most extensive community, encompassing 315 authors, are mainly affiliated with the Universidade Federal de Minas Gerais.

**Figure 10:**
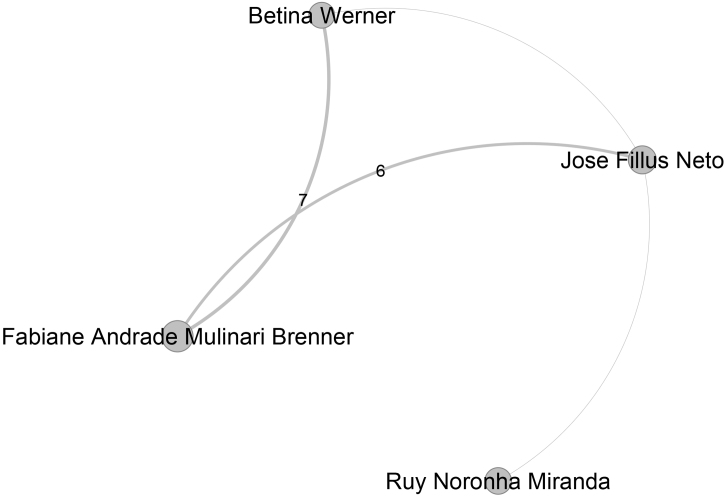
Top authors of the nineth largest community, which contains 297 authors, are primary affiliated with the Universidade Federal do Paraná.

Regarding the communities, the authors who are most relevant within the largest communities stand out. Aiming to focus the attention just on the main authors of each community, Figs. 2 to 10 present just authors who have 20 or more publications. Even the major networks have core members, demonstrating a pattern of close collaboration that has developed over time. The largest community, presented in Fig. 2, with 831 members, is led by the author Rubem David Azulay (Pos = 1), an emeritus professor at the Universidade Federal do Rio de Janeiro. This community contains 7.7% of the Journal authors and 22% of the authors in the ranking. The second largest community, shown in Fig. 3, comprises 549 authors and is led by Luna Azulay Abulafia (Pos = 21) from Universidade Estadual do Rio de Janeiro. The third largest community, displayed in Fig. 4, with 534 members, is led by Hiram Larangeira de Almeida Junior (Pos = 4), from Universidade Federal de Pelotas and Universidade Católica de Pelotas, and Renan Rangel Bonamigo (Pos = 5) from Universidade Federal do Rio Grande do Sul in Porto Alegre. The fourth largest community, displayed in Fig. 5, with 422 members, is led by Tania Ferreira Cestari (Pos = 30) from Universidade Federal do Rio Grande do Sul. Helio Amante Miot (Pos = 2) and Silvio Alencar Marques, both from Universidade Estadual Paulista, lead the fifth-largest community, which is composed of 349 authors, as presented in Fig. 6.

The sixth largest community, presented in Fig. 7, contains 334 authors, is led by Neusa Yuriko Sakai Valente (Pos = 7), from Universidade de São Paulo. Fig. 8 presents the seventh-largest community, which contains 320 authors, and is led by Rosana Lazzarini (Pos = 28), from Santa Casa de São Paulo. Bernardo Gontijo (Pos = 6), from Universidade Federal de Minas Gerais, leads the eighth largest community, which contains 315 authors and is presented in Fig. 9. The ninth largest community, which has 297 authors, is depicted in Fig. 10 and is headed by Fabiane Andrade Mulinari Brenner, from Universidade Federal do Paraná. These nine main communities encompass 3,897 authors, representing 36% of the ABD’s authors.

Although there is an international author, Cesare Massone (Pos = 283), among the top five in betweenness centrality, international authors are notably absent from the top 50 in the 100-year ranking. Despite its leadership in Latin America, the journal has historically been focused on Brazilian research, with limited interactions between Brazilian dermatologists and international collaborators. Additionally, publications by foreign authors gained prominence in earlier periods. This can be attributed to a relatively recent shift in editorial policy, implemented 20 years ago, aimed at broadening the base of national and international authors and reviewers. This effort was further supported by the journal’s indexing in LILACS since 1981, SciELO since 2003, and PubMed/Medline since 2009, which enhanced its visibility and accessibility to a wider audience.^1^

To evaluate the impact of editorial policy changes and initiatives aimed at enhancing the international visibility of ABD, the authors conducted a separate analysis focusing on the last 20 years (Table 2). This analysis identified one international researcher among the top 50 ranked authors: Toshiyuki Yamamoto (Pos = 29) from Japan, recognized as a notable foreign contributor. Despite this inclusion, the ABD ranking remains predominantly composed of Brazilian researchers, who continue to regard the journal as a key platform for disseminating their scientific work.

**Table 2:** Ranking of the top 50 authors over the past 20-years (2005–2024).

Pos	Name	Pub	Deg	BC	PR
1	Helio Amante Miot	139	257	0.059630	0.004793
2	Silvio Alencar Marques	85	87	0.017733	0.002225
3	Renan Rangel Bonamigo	75	169	0.031555	0.003063
4	Hiram Larangeira de Almeida Junior	70	122	0.016010	0.002825
5	Neusa Yuriko Sakai Valente	67	114	0.013105	0.002310
6	Paulo Ricardo Criado	51	112	0.029076	0.002058
7	Bernardo Gontijo	51	62	0.018284	0.001357
8	Everton Carlos Siviero do Vale	49	35	0.003428	0.001061
9	Mariangela Esther Alencar Marques	43	79	0.003543	0.001525
10	Milvia Maria Simoes e Silva Enokihara	42	87	0.009567	0.001639
11	Antonio Pedro Mendes Schettini	42	76	0.006204	0.001569
12	Sinesio Talhari	39	71	0.015011	0.001509
13	Lucia Martins Diniz	38	53	0.008012	0.001425
14	Rosana Lazzarini	36	87	0.008908	0.001317
15	Izelda Maria Carvalho Costa	36	52	0.006545	0.001131
16	John Verrinder Veasey	35	66	0.004808	0.001182
17	Luciana Patricia Fernandes Abbade	34	64	0.007285	0.001182
18	Alexandre Carlos Gripp	32	81	0.010977	0.001210
19	Fabiane Andrade Mulinari Brenner	31	74	0.015064	0.001297
20	Juliano Vilaverde Schmitt	31	73	0.008647	0.001194
21	Carolina Talhari	30	72	0.037232	0.001278
22	Monica Santos	30	48	0.002046	0.001116
23	Luna Azulay Abulafia	29	140	0.043686	0.001701
24	Juan Manuel Pineiro Maceira	29	89	0.014520	0.001363
25	Carlos Baptista Barcaui	29	83	0.024486	0.001225
26	Adriana Maria Porro	29	69	0.008886	0.001118
27	Rute Facchini Lellis	29	62	0.003728	0.001027
28	Marilda Aparecida Milanez Morgado de Abreu	27	61	0.007407	0.001243
29	Toshiyuki Yamamoto	27	18	0.000003	0.000653
30	Maraya De Jesus Semblano Bittencourt	26	58	0.005795	0.001145
31	Fred Bernardes Filho	24	67	0.006301	0.001279
32	Tania Ferreira Cestari	23	110	0.016329	0.001378
33	Valeria Aoki	23	86	0.011628	0.001230
34	Ana Maria Roselino	23	62	0.013043	0.001070
35	Mario Cezar Pires	22	94	0.016492	0.001217
36	Zilda Najjar Prado de Oliveira	22	87	0.006758	0.001175
37	Betina Werner	22	44	0.006083	0.000779
38	Priscila Kakizaki	22	37	0.000678	0.000771
39	Magda Blessmann Weber	21	84	0.007522	0.001172
40	Maria De Fatima Guimaraes Scotelaro Alves	21	51	0.001566	0.000846
41	Luciane Donida Bartoli Miot	21	40	0.000629	0.000718
42	Anna Carolina Miola	21	38	0.000470	0.000672
43	Flavia Vasques Bittencourt	20	52	0.011120	0.000792
44	Mariana de Figueiredo Silva Hafner	20	31	0.000248	0.000625
45	Vidal Haddad Junior	20	26	0.002144	0.000466
46	Gunter Hans Filho	19	70	0.007991	0.000964
47	Sergio Henrique Hirata	19	65	0.019640	0.000893
48	Carlos D'Apparecida Santos Machado Filho	19	53	0.004576	0.000903
49	Flavia Regina Ferreira	19	32	0.003030	0.000786
50	Rodrigo Pereira Duquia	19	30	0.001435	0.000764

The limitations of this study include the lack of consideration for authorship order (e.g., first or last author), article type (e.g., case report, review, editorial, or full article), and article impact, as measured by citation rate. These limitations highlight gaps that should be addressed in future research by examining the influence of these factors on authors, research communities, and their respective topics of study.

Finally, following open science practices, the data generated in this work, along with high-resolution images of both the complete network and the main co-authorship communities, are available at https://github.com/sandrocamargo/publications/tree/main/abd25.

## Conclusions

This study analyzed the century-long history of the ABD journal (1925–2024), encompassing 99 volumes and 6,299 articles authored by 10,829 distinct contributors. A ranking of the top 50 authors was constructed based on metrics including publication count, collaboration degree, betweenness centrality, and PageRank. Among the findings, Rubem David Azulay was recognized as the author with the highest number of publications, while Helio Amante Miot emerged as the most influential contributor across the other metrics. Moreover, 73.64% of all authors published only once in the journal. The study also mapped the main co-authorship communities, detailing their size, key members, and institutional affiliations.

These results provide a detailed overview of the journal’s historical contributions, recognizing individuals and communities that have significantly shaped its legacy over nearly a century.

## Financial support

None declared.

## Authors’ contributions

Helena Cargnelutti Grimaldi: Critical review of important intellectual content; interpretation of data; effective participation in the research guidance; final approval of the final version of the manuscript.

Sandro da Silva Camargo: The study concept and design; data collection and analysis; writing of the manuscript; final approval of the final version of the manuscript.

## Conflicts of interest

None declared.
